# Whole-Genome Sequencing and Concordance Between Antimicrobial Susceptibility Genotypes and Phenotypes of Bacterial Isolates Associated with Bovine Respiratory Disease

**DOI:** 10.1534/g3.117.1137

**Published:** 2017-07-26

**Authors:** Joseph R. Owen, Noelle Noyes, Amy E. Young, Daniel J. Prince, Patricia C. Blanchard, Terry W. Lehenbauer, Sharif S. Aly, Jessica H. Davis, Sean M. O’Rourke, Zaid Abdo, Keith Belk, Michael R. Miller, Paul Morley, Alison L. Van Eenennaam

**Affiliations:** * Department of Animal Science, University of California, Davis, California 95616; † Department of Microbiology, Immunology, and Pathology, College of Veterinary Medicine and Biomedical Sciences, Colorado State University, Fort Collins, Colorado 80523; ‡ California Animal Health and Food Safety Laboratory, School of Veterinary Medicine, University of California, Davis, Tulare, California 93274; § Veterinary Medicine Teaching and Research Center, School of Veterinary Medicine, University of California, Davis, Tulare, California 93274; ** Department of Population Health and Reproduction, School of Veterinary Medicine, University of California, Davis, California 95616; †† Department of Animal Sciences, College of Agricultural Sciences, Colorado State University, Fort Collins, Colorado 80523; ‡‡ Department of Clinical Sciences, College of Veterinary Medicine and Biomedical Sciences, Colorado State University, Fort Collins, Colorado 80523

**Keywords:** *Histophilus somni*, *Mycoplasma bovis*, *Mannheimia haemolytica*, *Pasteurella multocida*

## Abstract

Extended laboratory culture and antimicrobial susceptibility testing timelines hinder rapid species identification and susceptibility profiling of bacterial pathogens associated with bovine respiratory disease, the most prevalent cause of cattle mortality in the United States. Whole-genome sequencing offers a culture-independent alternative to current bacterial identification methods, but requires a library of bacterial reference genomes for comparison. To contribute new bacterial genome assemblies and evaluate genetic diversity and variation in antimicrobial resistance genotypes, whole-genome sequencing was performed on bovine respiratory disease–associated bacterial isolates (*Histophilus somni*, *Mycoplasma bovis*, *Mannheimia haemolytica*, and *Pasteurella multocida*) from dairy and beef cattle. One hundred genomically distinct assemblies were added to the NCBI database, doubling the available genomic sequences for these four species. Computer-based methods identified 11 predicted antimicrobial resistance genes in three species, with none being detected in *M. bovis*. While computer-based analysis can identify antibiotic resistance genes within whole-genome sequences (genotype), it may not predict the actual antimicrobial resistance observed in a living organism (phenotype). Antimicrobial susceptibility testing on 64 *H. somni*, *M. haemolytica*, and *P. multocida* isolates had an overall concordance rate between genotype and phenotypic resistance to the associated class of antimicrobials of 72.7% (*P* < 0.001), showing substantial discordance. Concordance rates varied greatly among different antimicrobial, antibiotic resistance gene, and bacterial species combinations. This suggests that antimicrobial susceptibility phenotypes are needed to complement genomically predicted antibiotic resistance gene genotypes to better understand how the presence of antibiotic resistance genes within a given bacterial species could potentially impact optimal bovine respiratory disease treatment and morbidity/mortality outcomes.

Historically, bacterial pathogens were identified by biochemical, antigen, or PCR-based methods, with results taking from days to weeks. Culture-independent diagnostic testing (CIDT), typically used to identify slow-growing agents or those that cannot be cultured *in vitro* (Janda and Abbot 2014), is gaining attention due to the availability of whole-genome sequence (WGS) data. WGS can reveal a pathogen’s identity and uncover other clinically useful information typically garnered through multiple diagnostic tests (Gilmour *et al.* 2013).

Employment of WGS for pathogen identification in *Salmonella* (Mohammed and Cormican 2015), *Klebsiella* *pneumoniae* (Marsh *et al.* 2015), *Neisseria* *meningitidis* (Mustapha *et al.* 2015), and *Escherichia coli* (Berenger *et al.* 2015) outbreaks demonstrated the effectiveness of this approach, but highlighted significant limitations due to the paucity of published pathogen genome sequences. It was recognized that broad sampling and establishing a global genomic database from isolates would be required to understand the genetic diversity and distribution of traits within a pathogen population (Mutreja *et al.* 2011; Koser *et al.* 2014).

CIDT could be useful in characterizing pathogens associated with bovine respiratory disease (BRD) complex, the most prevalent and costly cause of mortality for cattle in the United States (Chirase and Green 2000; USDA 2011a). Several bacterial pathogens are associated with BRD, with *Histophilus somni*, *Mycoplasma bovis*, *Mannheimia haemolytica*, and *Pasteurella multocida* being the most common (Griffin *et al.* 2010). Complete bacterial genomes are available for two strains of *H. somni*, five of *M. bovis*, 10 of *M. haemolytica*, and 11 of *P. multocida* (Table 1).

**Table 1:** Known reference sequences for the species sequenced in this study

Strain	GenBank Accession	Publication
2336	GCA_000019405.1	Siddaramappa *et al.* (2011)
129PT	GCA_000011785.1	Challacombe *et al.* (2007)
Hubei1	GCA_000219375.1	Li *et al.* (2011)
HB0801	GCA_000270525.1	Qi *et al.* (2012)
CQ-W70	GCA_000696015.1	None
NM2012	GCA_000183385.1	Wise *et al.* (2010)
PG45	GCA_001043135.1	None
M42548	GCA_000376645.1	Eidam *et al.* (2013)
89010807N_lktA	GCA_000963675.1	Heaton *et al.* (2015)
D153	GCA_000422145.1	Hauglund *et al.* (2013)
D171	GCA_000427275.1	Hauglund *et al.* (2015a)
D174	GCA_000422095.1	Hauglund *et al.* (2015b)
89010807N	GCA_000963635.1	Heaton *et al.* (2015)
USDA-ARS-SAM-185	GCA_000349785.1	Harhay *et al.* (2013)
USDA-ARS-USMARC-183	GCA_000349765.1	Harhay *et al.* (2013)
USDA-ARS-USMARC-184	GCA_000819525.1	None
USMARC_2286	GCA_000439735.1	Koren *et al.* (2013)
36950	GCA_000234745.1	Michael *et al.* (2011)
3480	GCA_000259545.1	None
ATCC_43137	GCA_000754275.1	Davenport *et al.* (2014)
HB01	GCA_001663095.1	Peng *et al.* (2016)
HB03	GCA_000512395.1	None
HN06	GCA_000255915.1	Liu *et al.* (2012)
OH1907	GCA_000973565.1	None
OH4807	GCA_000973525.1	None
PMTB2.1	GCA_001578435.1	None
Pm-3	GCA_001721885.1	None
Pm70	GCA_000006825.1	May *et al.* (2001)

It is important to consider antimicrobial resistance and the spread of antimicrobial resistance genes (ARG) in treatment decisions associated with common diseases such as BRD (Koser *et al.* 2014). Understanding the epidemiology of antimicrobial resistance in BRD pathogens in healthy cattle, as well as treated and untreated sick cattle, is crucial to understanding the role of resistance in BRD treatment failure. In addition, the increasing use of WGS in antimicrobial resistance surveillance necessitates a better understanding of the degree of concordance between phenotypic susceptibility and resistance genotype.

The primary objective of this study was to perform WGS on bacterial isolates collected from untreated BRD-affected beef and dairy cattle, and unaffected dairy cattle, to contribute additional bacterial genome sequences that could be used for CIDT in the future. Secondary study objectives were to perform bioinformatic analyses and susceptibility testing to analyze concordance between phenotypic susceptibility and resistance genotype. Identification of distinguishing genomic characteristics, such as ARG, of different strains of the major BRD pathogens may facilitate a better understanding of these pathogens and their host interactions with the goal of identifying improved treatment methods specific to the host species, ARG genotype, and pathogen combination.

## Materials and Methods

### Cattle sampling

Personnel at a northern California feedlot and a central California dairy calf nursery cared for the animals used in this study. In the calf nursery, preweaned Holstein dairy calves 22–60 d of age, who had never been treated for BRD nor treated with antimicrobials for any reason within the previous 10 d, were enrolled in a matched case-control study between July 2011 and January 2012, as described previously (Love *et al.* 2014; Neibergs *et al.* 2014). Upon morning observation, BRD suspect calves and matched adjacent control calves were scored using a clinical scoring system that assigns numerical values from 0 to 3 based on severity of symptoms for rectal temperature, cough, nasal discharge, ocular discharge, ear or head tilt or droop, and fecal consistency, with a score of ≥5 classified as a case (McGuirk 2008). Guarded deep nasopharyngeal swabs were collected from cases and control calves and placed into 1 ml of bacterial transport media (*Brucella* broth with 15% glycerol) for the detection of bacteria as described (Love *et al.* 2014). All samples were stored on wet ice in the dark until completion of sampling and delivery to the lab for aerobic bacteria and *Mycoplasma* culturing (Fulton and Confer 2012). Similar procedures were followed for beef cattle diagnosed as BRD cases on a California feedlot. Sampling was performed in compliance with approved Institutional Animal Care and Use protocols at the University of California, Davis (protocols 16431 and 17974).

### Bacterial isolates

Aerobic cultures from dairy calves were set up on sheep blood agar and chocolate agar plates and incubated at 37° in 7% CO_2_ for 2–4 d. Swabs for *Mycoplasma* isolation were swished in *Mycoplasma* D broth and incubated aerobically at 37° for 2–3 d, then plated to *Mycoplasma* N plates and incubated at 37° in 7% CO_2_ for up to 7 d. Bacteria from dairy cattle were isolated by morphology and subjected to testing for species identification at the California Animal Health and Food Safety Laboratory System Tulare laboratory. Urease detection, triple sugar iron agar, oxidase, indole production, hemolysis on sheep blood agar, and Gram stain testing were performed on *M. haemolytica*, *P. multocida*, and *H. somni* isolates. β-Galactosidase testing was performed on *P. multocida* and *M. haemolytica* and trehalose fermentation testing was performed on all *M. haemolytica* samples. Catalase and motility were also performed on *H. somni* isolates. *M. bovis* samples were confirmed as *Mycoplasma* by colony morphology, Dienes stain, and digitonin susceptibility prior to identification.

Ninety-four isolates of the four target organisms were frozen at −80°. Isolates were preferentially selected from cases rather than controls, generally from samples with moderate to large numbers of the organism in pure or predominant culture, and selection was spread out over the 6 months of the study. Four nonhemolytic *M. haemolytica* isolates were identified and removed from further analysis. *Mycoplasma* spp. were picked based on rapid growth and classic appearance for *M. bovis*, with only one morphotype present to prevent mixed cultures. In total, 74 case samples and 20 control samples were submitted for sequencing: *H. somni* (*n* = 16; 13 cases, three controls), *M. bovis* (*n* = 20; 17 cases, three controls), *M. haemolytica* (*n* = 28; 20 cases, eight controls), and *P. multocida* (*n* = 30; 24 cases, six controls). Species-identified isolates from dairy cattle were streaked onto various media and cultured at 37° for 1–5 d, depending on the species (Table 2). A single colony was added to 5 ml of PPLO broth for *M. bovis* isolates and BHI broth for the other three species, and incubated at 37° for 1–5 d prior to DNA isolation.

**Table 2:** Growth conditions for each of the four species used during this study

Species	Plate Media	Days for Growth	Broth Media	Days for Growth	Shaker	Environment
*Histophilus somni*	Columbia agar	1–2	BHI	1–2	N/A	Anaerobic
*Mycoplasma bovis*	PPLO	5	PPLO	5	N/A	5% CO_2_
*Mannheimia haemolytica*	Sheep’s blood agar	1–2	BHI	1–2	250 rpm	Facultative
*Pasteurella multocida*	Sheep’s blood agar	1–2	BHI	1–2	250 rpm	Facultative

BHI, brain–heart infusion; PPLO, pleuropneumonia-like organism.

One swab from each of the beef cattle sampled was plated on sheep blood agar, Columbia agar, and PPLO agar plates, and cultured at 37° for 1–5 d. Ten single colonies were isolated by morphology, replated, and cultured as described. A total of 10 samples were submitted from beef feedlot cattle: *M. haemolytica* (*n* = 2; two cases) and *P. multocida* (*n* = 8; eight cases).

### DNA isolation

A 2.5 ml culture was centrifuged at 13,000 rpm for 5 min for *H. somni*, *M. haemolytica*, and *P. multocida*, and for 20 min for *M. bovis*. The supernatant was discarded and the pellet was resuspended in 200 μl of PBS. DNA was extracted using the QIAamp Cador Pathogen Mini Kit (Qiagen Inc., Valencia, CA) according to the manufacturer’s instructions.

### PCR identification

PCR with species-specific primers was used to identify the four bacterial species from isolates obtained from beef cattle, and to confirm the biochemical (aerobic) and morphologic (*Mycoplasma*) identification of the isolates from the dairy cattle. Primers were designed in Primer3 (Koressaar and Remm 2007; Untergrasser *et al.* 2012) to targeted genes specific to each species (Table 3). PCR protocols for *M. haemolytica* (Alexander *et al.* 2008) and *P. multocida* (Ullah *et al.* 2009) were performed as described previously. Protocols for *H. somni* and *M. bovis* were developed during this study. PCR was performed on an Eppendorf Mastercycler (Eppendorf, Hamburg, Germany) with 1× GoTAQ Green Master Mix (Promega Biosciences LLC, San Luis Obispo, CA), 10 μM of each primer and 10 ng of DNA for 5 min at 95°, 35 cycles of 30 sec at 95°, 30 sec at 56°, and 45 sec at 72°, followed by 10 min at 72°. Products were visualized on a 1.5% agarose gel using a ChemiDoc-It^TS2^ Imager (UVP, LLC, Upland, CA), purified using the QIAquick PCR Purification Kit (Qiagen), Sanger sequenced (www.davissequencing.com) and aligned to their respective reference genomes using Bowtie2–default v2.2.8 (Langmead and Salzberg 2012) to verify that the appropriate target regions were amplified.

**Table 3:** Primers and PCR amplification protocols used in this study

Species	Primer	Sequence (5′-3′)	Tm (°)
*Histophilus somni*	Lob1F	AAATGGAAACACCCAACCAA	56.3
	Lob1R	CAAACGCTTGATACGCTCA	58.4
*Mycoplasma bovis*	MB528F	CATGCGAAAACACTGCAACT	58.4
	MB528R	CCACGCATTCAGAATTGAAA	56.3
*Mannheimia haemolytica* [Alexander *et al.* 2008]	LktF	GCAGGAGGTGATTATTAAAGTGG	61
	LktR	CAGCAGTTATTGTCATACCTGAAC	61.2
*Pasteurella multocida* [Ulhah *et al.* 2009]	KMT1SPG	GCTGTAAACGAACTCGCCAC	62.4
	KMT1T7	ATCCGCTATTTACCCAGTGG	60.4
Positive control	16SF	GCTAACTCCGTGCCAGCAG	64.5
	16SR	CGTGGACTACCAGGGTATCTAATC	65.6

### Library construction and sequencing

The PCR-confirmed DNA samples were sonicated on a Diagenode Bioruptor Pico Ultrasonicator (Diagenode Inc., Denville, NJ) to shear the DNA into 250 bp fragments. Next-generation sequencing libraries were created using the Kapa Genomic DNA Library Preparation kit (Kapa Biosystems, Wilmington, MA). The NEBNext Multiplex Oligos for Illumina kit (New England BioLabs Inc., Ipswich, MA) was used for indexing. The samples were sequenced (whole-genome shotgun, paired-end, 100 bp) on an Illumina HiSeq2500 sequencer at the Vincent J. Coates Genomics Sequencing Laboratory at UC Berkeley (supported by NIH S10 Instrumentation grants S10RR029668 and S10RR027303). A total of 104 isolates were whole-genome sequenced, along with five technical replicates.

### Read cleanup and de novo assembly

Raw reads were run through expHTS v0.0.Oct212015 (Street *et al.* 2015), which screens for contaminants using a mapped based approach, de-duplicates reads using Super-Deduper v2.0, performs quality trimming using Sickle v1.40, overlaps paired-end reads using Flash2 v2.2.00, and performs a quality analysis. Cleaned reads were then *de novo* assembled using the SPAdes assembler v3.5.0 (Bankevich *et al.* 2012) by running through the expHTS pipeline. Contigs were aligned to GenBank using BLAST (Altschul *et al.* 1990).

### Mapping assembly and genetic diversity

Available reference sequences were obtained from NCBI for each of the four species of interest (Table 1). Sequence reads from each isolate were aligned to their respective reference sequences using Bowtie2–default v2.2.8. SAM files were converted to BAM files and sorted using SAMtools v1.3 (Li *et al.* 2009). Consensus sequences were called using ANGSD v0.915 (Korneliussen *et al.* 2014) using -doFasta 3, which uses the base with the highest effective depth, and a minimum MAPQ score = 20. Average coverage for each consensus sequence was determined using SAMtools Depth v1.3. Consensus sequences were aligned and genetic distance trees, which were bootstrapped 100 times, were generated using SeaView v4.6.1 (Gouy *et al.* 2010). *M. haemolytica* sample sequences mapped to reference sequence USA-ARS-USMARC-183 were genotyped using previously described methods (Clawson *et al.* 2016). Statistical significance of genotype and the probability of being a case or control was assessed using the Fisher’s exact test.

### Antimicrobial resistance

Raw reads and *de novo* assembled contigs from each sample were aligned to the Resfinder database (v3-20-2017; Zankari *et al.* 2012) using Short Read Sequence Typing for Bacterial Pathogens (SRST2; Inouye *et al.* 2014) v0.2.0 and ABRicate v0.5-dev (https://github.com/tseemann/abricate), respectively, using default settings to identify resistance genes. For gene variants that confer resistance, *de novo* assembled contigs were aligned to the Comprehensive Antibiotic Resistance Database (CARD; Jia *et al.* 2017) variant model database using the BLAST-like alignment tool (BLAT; Kent 2002) v35, and visually inspected for resistance-conferring SNPs. Raw reads from each sample were aligned to the PlasmidFinder database (v3-23-2017; Carattolli *et al.* 2014) using the SRST2 v0.2.0 using default settings. The NCBI reference sequences were aligned to the Resfinder database using ABRicate.

### Susceptibility testing

Microdilution susceptibility testing of isolates was performed at the Kansas State Veterinary Diagnostic Laboratory using the Sensititre bovine/porcine plate (Thermo Scientific; plate code BOPO6F, Supplemental Material, Table S1), which tests for susceptibility to 18 drugs across nine antimicrobial classes: (i) β-lactam [Ampicillin (AMP), Ceftiofur (CEFT), Penicillin (PEN)]; (ii) tetracycline [Chlortetracycline (CHLOR), Oxytetracycline (OXY)]; (iii) fluoroquinolone [Danofloxacin (DAN), Enrofloxacin (ENRO)]; (iv) phenicol [Florfenicol (FLOR)]; (v) aminoglycoside [Gentamicin (GEN), Neomycin (NEO), Spectinomycin (SPEC)]; (vi) sulfonamide [Sulfadimethoxine (SUL)]; (vii) trimethoprim/sulfonamide [Trimethoprim-Sulfamethoxazole (TMS)]; (viii) macrolides-lincosamides (MLS) [Clindamycin (CLIN), Tilmicosin (TIL), Tulathromycin (TUL) Tylosin tartrate (TYL)]; and (ix) pleuromutilans [Tiamulin (TIA)] (Table 4). Breakpoints were chosen based on cattle-specific Clinical and Laboratory Standards Institute (CLSI) guidelines (CLSI 2013) when possible. CLSI swine-specific breakpoints were used for AMP and TIA, canine-specific breakpoints for CLIN and canine- and equine-specific breakpoints for GEN, and human-specific breakpoints for SUL and TMS. The CLSI breakpoints were not available for NEO and TYL, and therefore human-specific breakpoints from the National Antimicrobial Resistance Monitoring System were used (Table 4; FDA 2013). Wherever possible, breakpoints for the actual pathogens tested in this study were utilized. Based on these breakpoints, isolates were classified as susceptible, intermediate, or resistant. For analysis purposes, susceptible and intermediate isolates were considered to be susceptible. Crude prevalence of phenotypic resistance for each drug tested was calculated by bacterial species and across all three species. In addition, prevalence of multidrug resistant (MDR) phenotypes (defined as resistance to more than two antimicrobial drug classes) was calculated and MDR patterns were assessed.

**Table 4:** Mapping of identified resistance genes to antimicrobial drugs included on the broth microdilution phenotypic susceptibility panel

Resistance Gene	Antibiotic (Abbreviation)	Antibiotic Class	CLSI Approved Breakpoint	Breakpoint (mg/ml)		
				Susceptible	Intermediate	Resistant
*tetH*	Chlortetracycline (CHLOR)	Tetracycline	Yes	≤2	4	≥8
	Oxytetracycline (OXY)	Tetracycline	Yes	≤2	4	≥8
*aph3-Ia*	Neomycin (NEO)	Aminoglycoside	No*^a^*	≤2	4	≥8
*strA*	None tested	N/A	N/A	N/A	N/A	N/A
*strB*	None tested	N/A	N/A	N/A	N/A	N/A
*ROB-1*	Ampicillin (AMP)	β-Lactam	Yes*^b^*	≤0.5	1	≥2
	Ceftiofur (CEFT)	β-Lactam	Yes	≤2	4	≥8
	Penicillin (PEN)	β-Lactam	Yes	≤0.25	0.5	≥1
*sul2*	Sulfadimethoxine (SUL)	Sulfonamide	Yes*^c^*	≤256	—	≥512
	Trimethoprim-sulfamethoxazole (TMS)	Sulfonamide	Yes*^d^*	<0.5 and 9.5	1–2 and 19–38	4 and 76
*erm42/ermF*	Clindamycin (CLIN)	MLS	Yes*^e^*	≤0.5	1 - 2	≥4
	Tilmicosin (TIL)	MLS	Yes*^f^*	≤8	16	≥32
	Tulathromycin (TUL)	MLS	Yes	≤16	32	≥64
	Tylosin tartrate (TYL)	MLS	No*^g^*	≤8	16	≥32
*cat2*	Florfenicol (FLOR)	Phenicol	Yes	≤2	4	≥8
*floR*	Florfenicol (FLOR)	Phenicol	Yes	≤2	4	≥8
*dfrA14*	Trimethoprim-sulfamethoxazole (TMS)	TMS	Yes*^d^*	<0.5 and 9.5	1–2 and 19–38	4 and 76
None identified	Danofloxacin (DAN)	Fluoroquinolone	Yes*^c^*	≤0.25	—	—
None identified	Enrofloxacin (ENRO)	Fluoroquinolone	Yes	≤0.25	0.5–1	≥2
None identified	Gentamicin (GENT)	Aminoglycoside	Yes*^h^*	≤2	4	≥8
None identified	Spectinomycin (SPEC)	Aminoglycoside	Yes	≤32	64	≥128
None identified	Tiamulin (TIA)	Pleuromutilin	Yes*^i^*	≤16	—	≥32

MLS, macrolide-lincosamide-streptogramin.

a For Neomycin, we used NARMS 2011 breakpoints for aminoglyosides.

b For Ampicillin, we used CLSI breakpoints for swine that were validated for the following bacteria: *Actinobacillus pleuropneumonia*e and *P. multocida*.

c For Danofloxacin, we used CLSI breakpoints for cattle, but only validated for the following bacteria: *M. haemolytica* and *P. multocida*.

d For Sulfadimethoxine and TMS, we used CLSI breakpoints for humans with systemic disease and validated in *Enterobacteriaceae*.

e For Clindamycin, we used CLSI breakpoints for canines that were validated for the following bacteria: *Staphylcoccus* spp. and β-hemolytic *Streptococci*.

f For Tilmicosin, we used CLSI breakpoints for cattle, but only validated for the following bacteria: *M. haemolytica*.

g For Tylosin, we used the same breakpoints as in the following document: https://www.ars.usda.gov/SP2UserFiles/Place/60400520/NARMS/ABXEntero.pdf.

h For Gentamicin, we used canine and equine breakpoints validated in *Pseudomonas aeruginosa* and *Enterobacteriaceae* and the equine breakpoint for *Actinobacillus* spp.

i For Tiamulin, we used CLSI breakpoints for swine that were validated for the following bacteria: *Actinobacillus pleuropneumoniae*.

In order to compare phenotypic and genotypic susceptibility results, identified resistance genes were mapped to the antimicrobial drug classes to which they are known to confer resistance (Table 4). Proportion of isolates with concordant and discordant genotype–phenotype results were calculated for each antimicrobial drug and organism.

Statistical significance of discordance was assessed using the mid-*P* exact for binomial paired data with a two-tailed *P*-value (Edwards 1948), unless there were at least 20 discordant observations or the marginal counts were equal to zero, in which case McNemar’s test with continuity correction was used (Fagerland *et al.* 2013). If there was a lack of observations in any of the genotype–phenotype concordance–discordance combinations, 0.5 was added to the count in order to enable calculation of the test statistic. OpenEpi was used for all calculations (Dean *et al.* 2013). A two-tailed *P*-value was used and the α level was 0.05.

### Data availability

Sequence reads are available in the NCBI Sequence Read Archive as Bioproject PRJNA306895 and SRA accession number SRP095705. *De novo* assemblies are available as NCBI whole genome shotgun sequences (accession numbers: LQCX01-LQGS01). GenBank accession numbers are SRR5133037–SRR5133136. Additional supporting data are provided in Figure S1, Figure S2, Figure S3, Figure S4, Figure S5, Figure S6, File S1, Table S1, Table S2, Table S3, Table S4, and Table S5.

## Results

### Sequencing and assembly

Laboratory-confirmed isolates of *H. somni* (*n* = 16; 13 cases, three controls), *M. bovis* (*n* = 20; 17 cases, three controls), *M. haemolytica* (*n* = 28; 20 cases, eight controls), and *P. multocida* (*n* = 30; 24 cases, six controls) from dairy cattle; and *M. haemolytica* (*n* = 2; two cases) and *P. multocida* (*n* = 8; eight cases) from beef cattle were sequenced, and raw reads were processed and assembled (Table 5 and Table S2). *Staphylococcus aureus* contamination was identified in one of the *P. multocida* samples isolated from an affected dairy calf and from two of the beef cattle samples, as well as in one of the beef cattle *M. haemolytica* samples. These samples were excluded from further analysis. A total of 100 sequences—16 *H. somni* (all dairy), 20 *M. bovis* (all dairy), 29 *M. haemolytica* (28 dairy, one beef), and 35 *P. multocida* (29 dairy, six beef)—were selected for further analysis. Raw reads from these 100 samples were mapped to each of their respective reference sequences and subjected to further analysis.

**Table 5:** Average assembly statistics for each species sequenced in this study from *de novo* assembled contigs

Species	Contig	Coverage	Total Length	N50	Min. Contig	Max. Contig
*Histophilus somni*	77	62.46	2,136,280	89,438	1094	294,083
*Mycoplasma bovis*	74	72.41	943,236	27,508	1061	59,658
*Mannheimia haemolytica*	105	35.68	2,637,528	70,779	1068	282,147
*Pasteurella multocida*	33	29.33	2,317,940	171,866	1099	469,530

Total length is the total of all contig lengths; N50 is 50% of the mass of distribution of the contig lengths; Min. contig and Max. contig are the length of the shortest and longest contigs in each assembly.

Of the 29 *M. haemolytica* samples, 22 were classified as genotype 2 and the other seven were classified as genotype 1, as recently described by Clawson *et al.* (2016). Of the 22 samples classified as genotype 2, 19 were obtained from cases (86.4%; *P* < 0.01); while of the seven samples classified as genotype 1, five were obtained from controls (71.4%; *P* < 0.01). Samples classified as genotype 2 have largely been associated with those taken from BRD-affected animals, while samples classified as genotype 1 have been associated with nonclinical isolates of *M. haemolytica*. There have been some genotype 1 samples obtained from BRD-affected animals, as well as genotype 2 samples obtained from non-BRD–affected animals (Clawson *et al.* 2016).

**Figure 1:**
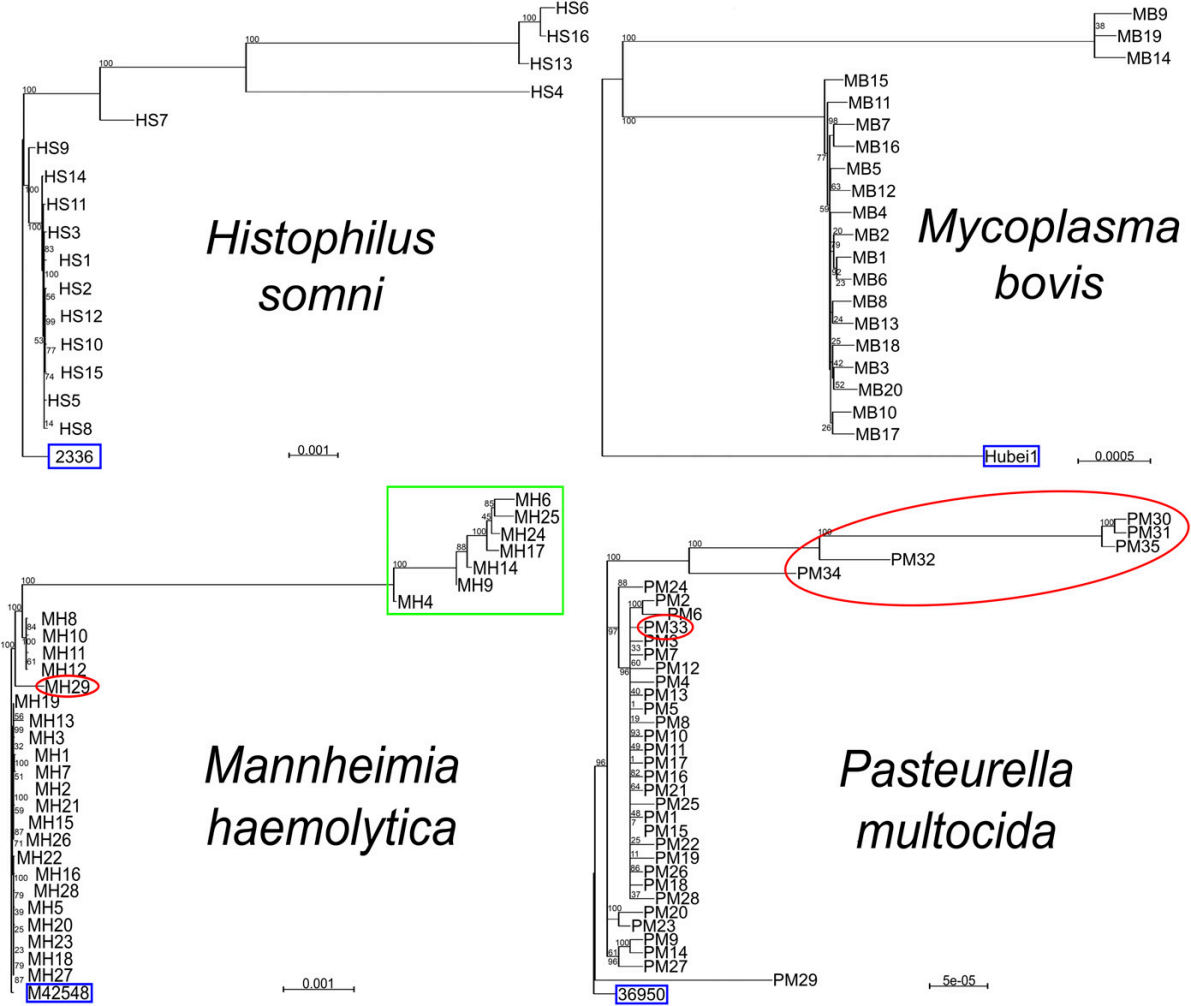
Genetic distance trees from mapped assemblies of the four bacterial species, *Histophilus somni* aligned to reference sequence 2336, *Mycoplasma bovis* aligned to Hubei1, *Mannheimia haemolytica* aligned to reference sequence M42548, and *Pasteurella multocida* aligned to reference sequence 36950. Red circle, beef cattle; blue box, reference sequence; green box, *M. haemolytica* genotype 1 (Clawson *et al.* 2016). Scale bar represents number of substitutions per site.

Genetic distance trees were generated to visualize the genetic distance between the genomic sequences and mapped assemblies. Overall, the sequence assemblies for the samples collected in this study increased the genetic diversity for available WGS for three out of the four species analyzed, *H. somni*, *M. bovis*, and *P. multocida* (Figure 1). Five of the *H. somni* samples contained sequences that were genomically distant from both available reference sequences, while the other 11 samples were genomically distant from reference sequence 129PT (Figure S1a). While the *M. bovis* samples showed little variation among themselves, all 20 samples were genomically distant from the five reference sequences (Figure S1b). The *M. haemolytica* sample population was the only species that revealed little genetic variation between the samples collected and the available reference sequences (Figure S1c). The seven samples classified as genotype 1 clustered together away from the other 22 samples (Figure S1c). The *P. multocida* sample population showed significant variation between the samples collected and six of the 11 available reference sequences (Figure S1d). For samples aligned to *P. multocida* reference sequences 3480, HN06, OH1905, OH4807, PMTB2.1, and Pm70, the study samples clustered together away from the reference sequence, showing a large distance between the sample group and the reference sequences. For the remaining five reference sequences, 36950, ATCC_43137, HB01, HB03, and Pm-3, there was little genetic diversity between the samples collected and the reference sequences.

### ARGs

**Figure 2:**
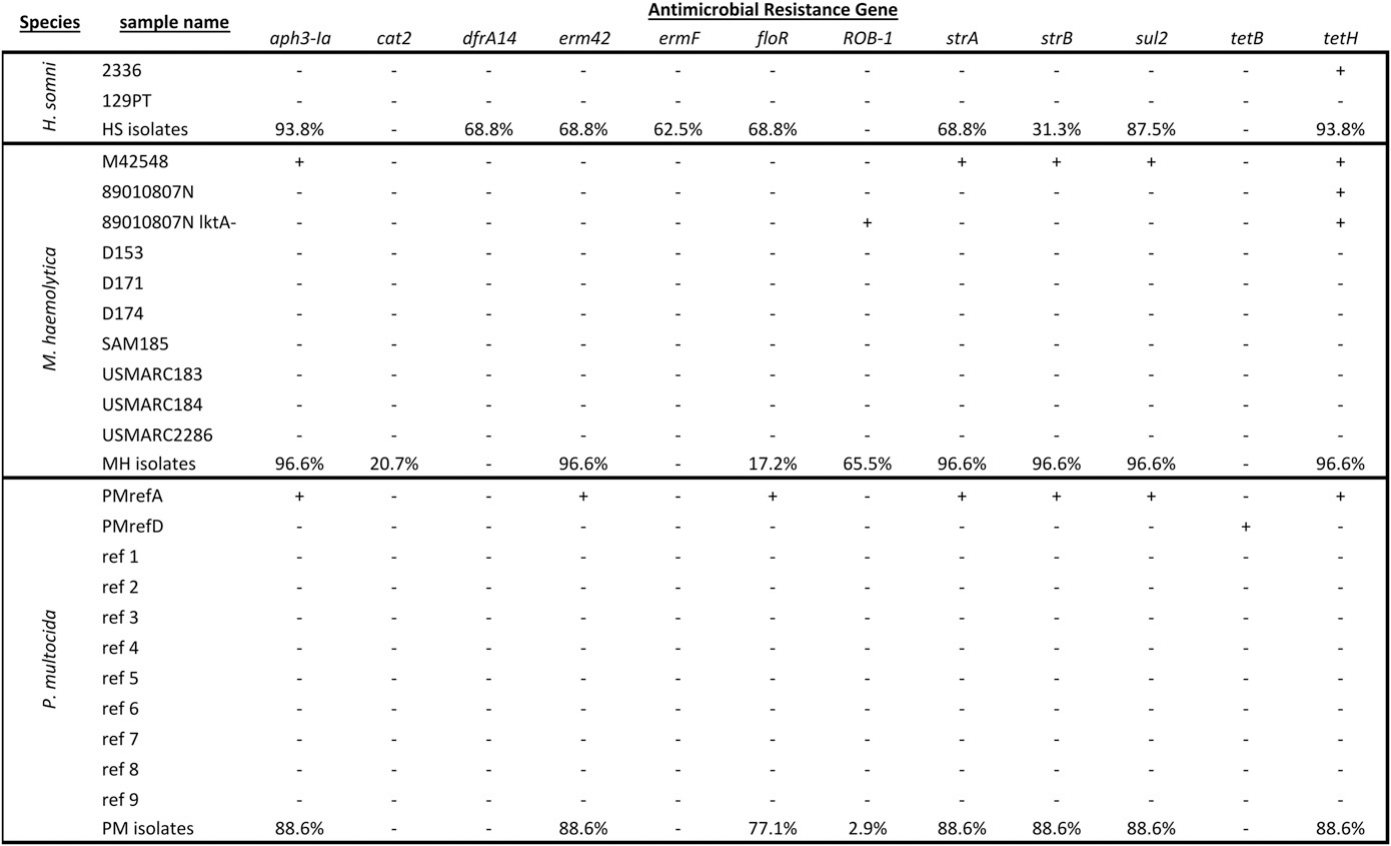
Prevalence of resistance genes among isolates of *Histophilus somni* (*N* = 16), *Mannheimia haemolytica* (*N* = 29), and *Pasteurella multocida* (*N* = 35), as well as available reference sequences. +, present; −, absent.

Raw read and *de novo* assembled contigs were aligned to the Resfinder database using SRST2 and ABRicate, respectively. Table 4 lists known ARG detected and the antimicrobial(s) to which the genes can confer resistance. Phenotypic antimicrobial susceptibility testing results were obtained for 64 isolates (13 *H. somni*, 26 *M. haemolytica*, and 25 *P. multocida*) for 18 antimicrobials from nine classes. The remaining three samples of *H. somni*, three samples of *M. haemolytica* and 10 samples of *P. multocida* failed to grow in the susceptibility testing positive control media and were excluded from analysis. Using *in silico* ARG prediction methods, 11 total ARG were detected, with two genes specific to *H. somni* isolates (*ermF* and *dfrA14*), two genes specific to *M. haemolytica* isolates (*ROB-1* and *cat2*), and the other seven detected in *H. somni*, *M. haemolytica*, and *P. multocida* (Figure 2). No ARG were detected in any of the *M. bovis* samples, and therefore, these samples were not subjected to susceptibility testing. Raw reads were mapped to the PlasmidFinder database using SRST2 to determine whether ARG present were on plasmids or integrated into the genome. No plasmids containing known ARG sequences were detected using this method. Additionally, alignment of *de novo* assembled contigs to the CARD protein variant model database did not reveal any variants in genes known to confer resistance to antimicrobials.

#### H. somni ARG:

*In silico* ARG identification was performed for the two *H. somni* reference sequences 2336 and 129PT (Figure 2). No ARG were predicted in reference sequence 129PT and only *tetH* was predicted in reference sequence 2336. Nine ARG were detected in the *H. somni* isolates (Figure S2). Two aminoglycoside resistance genes that confer resistance to the drugs KAN and NEO (*aph3-Ia*) and streptomycin (*strA*, *strB*) were detected. Two macrolide-lincosamide-streptogramin B (MLS_b_) resistance genes (*erm42* and *ermF*) were also detected, as was a phenicol resistance gene (*floR*), a sulfonamide resistance gene (*sul2*), a trimethoprim resistance gene (*dfrA14*), and a tetracycline resistance gene (*tetH*) (Figure 2). There was a 79.2% concordance rate between genes identified using raw reads and *de novo* assembled contigs.

#### M. haemolytica ARG:

Different ARG were identified across *M. haemolytica* reference sequences. The genes *aph3-Ia*, *strB*, *sul2*, *tetH* and the β-lactamase gene, *ROB-1*, were detected in reference sequence M42548; *ROB-1* was detected in reference sequence 89010807N_lktA; *tetH* was detected in reference sequence 89010807N_lktA and reference sequence 89010807N; no ARG were detected in any of the other available reference sequences (*n* = 7, Figure 2). Seven of the eight ARG detected in *M. haemolytica* isolates (*aph3-Ia*, *erm42*, *floR*, *strA*, *strB*, *sul2*, and *tetH*) were also found in *H. somni* and *P. multocida* (Figure 2). No ARG were detected in the only *M. haemolytica* sample sequenced from BRD-affected beef cattle (Figure S3). Two of the genes (*erm42* and *floR*) were not detected in any reference sequence but have been previously reported (Clawson *et al.* 2016). There was an 89.3% concordance rate between genes identified using raw reads and *de novo* assembled contigs. There was complete linkage of ARG *aph3-Ia*, *strA* and *strB* in 28 out of 29 samples, showing homology to the conserved backbone of ICE*Mh1* and ICE*Pmu1* (Figure S3). The presence of the conserved ICE backbone has been associated with samples classified as genotype 2. Here, 21 out of 22 samples classified as genotype 2 contained regions homologous to the ICE backbone and all seven samples classified as genotype 1 also contained regions homologous to the ICE backbone. Additionally, the phenicol resistance gene *cat2* was identified in six out of 29 samples. Six of the seven samples classified as genotype 1 contained the *cat2* ARG, while the other 22 samples, classified as genotype 2, did not contain the *cat2* ARG (Figure S3).

#### P. multocida ARG:

The available *P. multocida* reference sequences had similar ARG variation to that seen in the *M. haemolytica* reference sequences. All seven of the ARG (*tetH*, *aph3-Ia*, *erm42*, *strA*, *strB*, *floR*, and *sul2*) detected in the *P. multocida* samples were also detected in reference sequence 36950 (Michael *et al.* 2011). The gene *tetB* was detected in reference sequence HB01, but no ARG were detected in any of the other nine reference sequences (Figure 2). Seven of the 11 ARG were detected in the *P. multocida* samples originating from dairy cattle (Figure 2). Sequences from two samples obtained from beef cattle contained four and five of seven ARG, *tetH*, *aph3-Ia*, *strA*, *strB* and *tetH*, *aph3-Ia*, *erm42*, *strA*, *strB*, respectively, but there were no ARG detected in the other four samples obtained from beef cattle (Figure S4). One of the 35 samples contained the β-lactamase gene, *ROB-1*. There was a 76.5% concordance rate between genes identified using raw reads and *de novo* assembled contigs. Like *M. haemolytica* samples, there was complete linkage of ARG *aph3-Ia*, *strA* and *strB* in 31 out of 35 samples, showing homology to the conserved backbone of ICE*Mh1* and ICE*Pmu1*.

### Phenotypic susceptibility testing results

**Figure 3:**
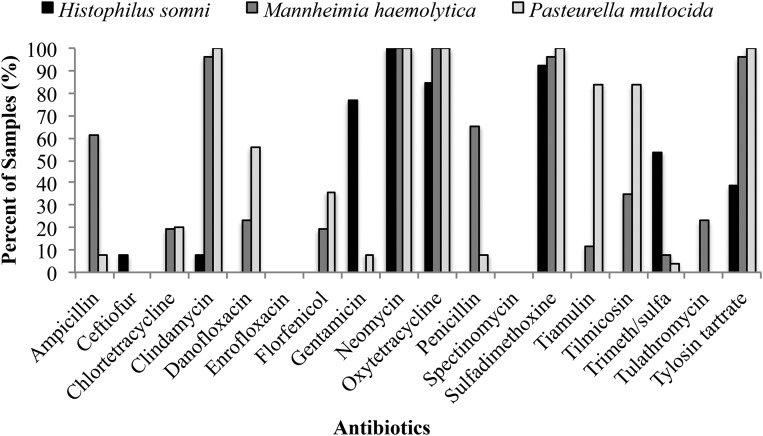
Prevalence of phenotypic resistance to all drugs tested among isolates of *Histophilus somni* (*N* = 13), *Mannheimia haemolytica* (*N* = 26), and *Pasteurella multocida* (*N* = 25).

All 64 isolates displayed phenotypic resistance to NEO at a resistant breakpoint of ≥32 μg/ml (File S1 and Table 6). All 25 of the *P. multocida* isolates were also resistant to TYL, OXY, SUL, and CLIN, while all 26 of the *M. haemolytica* were resistant to OXY (Figure 3). None of the isolates displayed phenotypic resistance to SPEC or ENRO, and a single *H. somni* isolate was resistant to CEFT (Table 6 and Table S3). *M. haemolytica* was the only organism that displayed resistance to TUL (five out of 26 isolates). None of the isolates from any species were susceptible to all drugs tested; 61 were MDR (defined as exhibiting phenotypic resistance to more than two antimicrobial classes tested) and the remaining three were resistant to only two classes of antimicrobials (Figure S5).

**Table 6:** Concordance of phenotypic and susceptibility results for all three organisms (64 isolates)

		Pheno: S		Pheno: R				
Class	Antibiotic	Geno:R	Geno:S	Geno:R	Geno:S	Discordant (%)	Concordant (%)	*P*-Value
β-Lactam	Ampicillin	4	42	15	3	11	89	0.73
β-Lactam	Ceftiofur	19	44	0	1	31	69	<0.001
β-Lactam	Penicillin	4	41	15	4	13	88	0.99
Tetracycline	Chlortetracycline	53	1	10	0	83	17	<0.001
Tetracycline	Oxytetracycline	1	1	62	0	2	98	0.50
Fluoroquinolone	Danofloxacin	0	44	0	20	31	69	<0.001
Fluoroquinolone	Enrofloxacin	0	64	0	0	0	100	0.32
Phenicol	Florfenicol	24	26	14	0	38	63	<0.001
Aminoglycoside	Gentamicin	0	52	0	12	19	81	0.002
Aminoglycoside	Neomycin	0	0	63	1	2	98	1.0
Aminoglycoside	Spectinomycin	0	64	0	0	0	100	0.37
Sulfonamide	Sulfadimethoxine	1	1	60	2	5	95	0.63
Trimeth/Sulf	TMS	33	21	7	3	56	44	<0.001
Pleuromutilin	Tiamulin	0	40	0	24	37	63	<0.001
MLS	Clindamycin	7	6	31	20	42	58	0.02
MLS	Tilmicosin	13	21	25	5	28	72	0.06
MLS	Tulathromycin	33	26	7	3	52	48	<0.001
MLS	Tylosin tartrate	5	4	33	22	42	58	<0.001

Pheno, phenotype; S, susceptible; R, resistant; Geno, genotype; Trimeth/Sulf, trimethoprim/sulfonamide; TMS, trimethoprim-sulfamethoxazole; MLS, macrolide-lincosamide-streptogramin.

### Genotype–phenotype concordance

Concordance rates for antimicrobial resistance genotype and phenotype varied widely for each antimicrobial drug (Table 6). Across all three organisms, ENRO and NEO exhibited 100% concordance, with all isolates displaying phenotypic susceptibility and none containing genes typically associated with resistance to these antimicrobials, *i.e.*, adenylyltransferases AAD(3″) and AAD(9) for SPEC and *parC*, *parE*, *gyrA*, and *gyrB* for ENRO. As with NEO, SPEC also exhibited high genotype–phenotype concordance (98%), but in contrast with NEO, this concordance was driven by presence of *aph3-Ia* and corresponding phenotypic resistance. Concordance to OXY was also very high across all three organisms; of the 64 isolates tested, 62 displayed phenotypic resistance to OXY and contained the *tetH* resistance gene. All three organisms exhibited statistically significant phenotype–genotype discordance for CHLOR and TIL (*P* < 0.05; Table S3, Table S4, and Table S5). CHLOR discordance was driven by phenotypic susceptibility in the presence of *tetH* (opposite the results for OXY). For *H. somni* and *P. multocida*, TIL discordance resulted from phenotypic susceptibility in the presence of *erm*F and/or *erm*42. Interestingly, *erm42* has previously been identified in MDR *P. multocida* isolates from cases of BRD, but has yet to be described in *H. somni* (Kadlec *et al.* 2011). Unlike TIL discordance in *H. somni* and *P. multocida*, discordance in *M. haemolytica* was due largely to phenotypic resistance without identification of a known macrolide resistance gene.

*H. somni* and *M. haemolytica* demonstrated significant discordance for CLIN, however this discordance was driven by different dynamics (Table S3 and Table S4). Within *H. somni*, discordance resulted from phenotypic susceptibility in the presence of both *erm*42 and *erm*F, whereas phenotypic resistance and a lack of any known macrolide resistance genes drove the discordance in *M. haemolytica*.

*H. somni* and *P. multocida* exhibited significant discordance for FLOR, which in both organisms resulted from phenotypic susceptibility despite presence of the *floR* resistance gene. In addition, TUL was significantly discordant in both *H. somni* and *P. multocida* (*P* < 0.05; Table S3, Table S4, and Table S5), in both cases due to presence of *erm42* and/or *ermF* without corresponding phenotypic resistance.

Phenotype and genotype results for DAN and TMS were significantly discordant in both *M. haemolytica* and *P. multocida* (*P* < 0.05; Table S4 and Table S5). For DAN, this discordance was due to phenotypic resistance without identification of a known danofloxacin resistance gene. Conversely, TMS discordance was driven by phenotypic susceptibility despite presence of the *sul2* gene.

In addition, *H. somni* exhibited significant discordance for GENT, with 10 out of 13 isolates displaying phenotypic resistance but none containing a resistance gene known to confer resistance to this drug. All 10 of these isolates contained *aph3-Ia*, but this gene is typically associated with resistance to the aminoglycosides kanamycin, NEO, and paromomycin within *Enterobacteriaceae* spp. (Livermore *et al.* 2001). The breakpoint used for GENT was developed for *Pseudomonas aeruginosa* and *Enterobacteriaceae* in canines and equids, and *Actinobacillus* spp. in equids (CLSI 2013), and it is unknown whether these breakpoints are appropriate for *H. somni* in cattle.

*M. haemolytica* isolates also displayed statistically significant discordance for both CEFT and TYL (*P* < 0.05; Table S4). In the case of TYL, 25 of the 26 isolates were phenotypically resistant, but only six of these 25 isolates contained *erm42*. CEFT discordance resulted from the fact that none of the isolates whose genomes contained *ROB-1* experienced CEFT resistance due to the presence of this β-lactamase gene.

*P. multocida* isolates were also significantly discordant for the pleuromutilin TIA (*P* < 0.05; Table S5), with 21 out of 25 isolates displaying phenotypic resistance despite lack of an identified pleuromutilin resistance gene within the isolates’ genomes.

**Figure 4:**
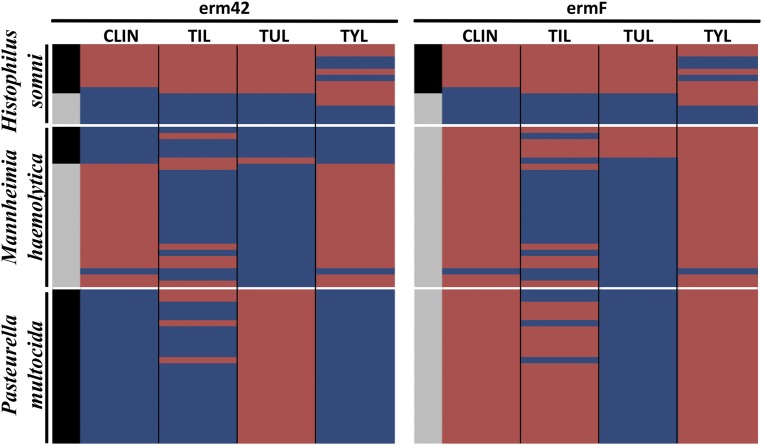
Heatmap showing concordance (blue) and discordance (red) between *erm42* and *ermF* presence (black) or absence (gray) and phenotypic resistance to the four macrolide drugs included in the susceptibility testing panel (columns), by isolate (rows) within organism. CLIN, clindamycin; TIL, tilmicosin; TUL, tulathromycin; TYL, tylosin tartrate.

Macrolide resistance is of particular concern, as BRD is typically treated with either tetracyclines or macrolides. We identified two MLS_b_ resistance genes within the 64 samples, *erm42* and *ermF*. The genotype–phenotype concordance results differed greatly by the combination of gene, bacteria, and macrolide drug in question, as illustrated in Figure 4. When averaged across all three bacterial species, the genotype–phenotype discordance rates were statistically significant for all four macrolides tested (Table 6). However, discordance rates and significance varied by organism (Table S3, Table S4, and Table S5). Additionally, there was not a strong correlation between the number of ARG detected in each sample and the number of antimicrobials that each sample was resistant to (Figure S6).

## Discussion

### Genome assembly and diversity

Additional WGS from BRD pathogens are needed to help understand genetic diversity and its role in pathogenesis, genotype–phenotype associations, and the relationship between genomic features and clinical disease. This study doubled the number of available genomic sequences for these four species. In addition to expanding current WGS databases, standardization of genome analysis methods for genomic epidemiological interpretation is important (Gilmour *et al.* 2013). As we have shown in this study, results obtained from *de novo* assemblies *vs.* consensus sequences constructed from mapping to reference genomes can be highly variable. This is demonstrated not only by the differences in genetic distances for each consensus sequence called when mapped to their respective reference sequences (Figure S1), but also by variability in ARG (Figure 2). However, there are many benefits to using a reference-based mapping approach, particularly when many samples need to be sequenced and analyzed. One such advantage is the computing power needed for consensus mapping is much lower as compared to *de novo* assembly and phylogenomic analyses using mapped assemblies yield similar results to those produced by *de novo* assemblies (Fitz-Gibbon *et al.* 2017). Genome assembly via mapping of reads to reference sequences enabled us to efficiently characterize variation within our study population, even if there is a slight bias toward the reference sequences (Figure S1).

Little variation was detected between samples mapped to each of the 10 *M. haemolytica* reference sequences. This is likely due to similarity in strains from cattle across the Midwest and California, as nine of the 10 reference sequences were obtained from cattle in the Midwest. Similarly, *P. multocida* samples clustered much more closely to reference sequences 3480, HN06, OH1905, OH4807, PMTB2.1, and Pm70, as each of these samples were obtained from cattle in California or the Midwest. In contrast, *P. multocida* samples in our study clustered more distantly from reference sequences 36950, ATCC_43137, HB01, HB03, and Pm-3, which were obtained from cattle in Germany or China. In this dataset, there was a clear distinction between *P. multocida* isolates collected from the beef and dairy cattle populations with the exception of one sample. However, it is important to note that only six of the 35 *P. multocida* samples analyzed were obtained from BRD-affected beef cattle. Further investigations with larger sample sizes from broader geographical ranges will be required to validate these observations.

### Predicting antimicrobial resistance

The ability to predict ARG *in silico* would have a significant impact on our ability to diagnose and treat pathogenic diseases and monitor the spread of ARG (Koser *et al.* 2014). In a study of animals previously treated with antimicrobials, resistance was identified in 72% of *M. haemolytica* and 50% of *P. multocida* isolates, with 13 ARG present in *H. somni* (Klima *et al.* 2014). Other studies in healthy cattle suggest that the prevalence of antimicrobial resistance in BRD-associated bacteria may be relatively low (Noyes *et al.* 2015). *M. haemolytica* and *P. multocida* harbor integrative and conjugative elements that enable the transfer of gene blocks between the species (Klima *et al.* 2014) and confer resistance to antimicrobials (Michael *et al.* 2011; Eidam *et al.* 2015). *M. bovis* lacks a cell wall and is resistant to drugs that target the cell wall of Gram-negative bacteria; to date no ARG have been found in any strain of *M. bovis* (Maunsell *et al.* 2011).

In this study, we detected 11 antibiotic resistance genes within the sample population, including three aminoglycoside resistance genes, two macrolide-lincosamide-streptogramin resistance genes, two phenicol and one each of β-lactam, sulfonamide, fluoroquinolone, tetracycline, and trimethoprim resistance genes. Tetracyclines and macrolides are often used as first-line agents in the treatment of BRD. The prevalence of *tetH* and *erm42* were relatively high in this population, and the presence of these ARG within BRD-associated bacterial isolates could render BRD treatment more challenging (Livermore *et al.* 2001). However, prevalence of resistance to antimicrobials typically used as second- or third-line treatments was relatively low, *i.e.*, *floR* and *cat2* for florfenicol. As a result, several alternative, commonly used antimicrobials for BRD treatment (USDA 2011b) could still be effective based on our findings.

Phenotypic antimicrobial resistance and the presence of ARG have both been demonstrated for *H. somni*, *M. haemolytica*, and *P. multocida* (Klima *et al.* 2014; DeDonder and Apley 2015). It is known that antimicrobials that target the cell wall, such as β-lactams, are not effective against *M. bovis* due to the lack of a cell wall. While we did not identify ARG in any of the *M. bovis* genomes in this study, phenotypic resistance within this organism has previously been demonstrated (Kawai *et al.* 2014; Kong *et al.* 2015). Each of the other three species has shown resistance to tetracyclines (Kehrenberg *et al.* 2001; D’Amours *et al.* 2011), which is the primary resistance gene found in *H. somni* samples in previous studies, but the majority of resistance genes previously found have been in *P. multocida* and *M. haemolytica*.

Previous studies have shown macrolide-lincosamide-streptogramin, kanamycin, NEO, and streptomycin resistance in *H. somni* samples (DeDonder and Apley 2015), but there were no ARG found in *H. somni* reference sequences other than *tetH*. However, recent studies have shown the presence of seven out of nine ARG found in *H. somni* samples during this study (Klima *et al.* 2014), showing the variation of ARG found in different strains. Recent studies showing the presence of known ARG, as well as phenotypic antimicrobial resistance, have primarily been done in diseased animals from feedlots around the Midwest, while the reference sequence samples were obtained from diseased animals in California. Our study shows a similarity in ARG to the recent study of samples obtained from feedlots in Texas, Nebraska, and Alberta, Canada (Klima *et al.* 2014). Importantly, eight of nine ARG found in *H. somni* and three of eight ARG found in *M. haemolytica* were not found in any of the previously available reference genome sequences.

It is important to note that there was slightly more ARG detected in each sample using raw reads compared to ARG detected using *de novo* assembled contigs. Using raw reads for gene detection has been shown to be superior to assembly-based approaches. This is primarily because assembly-based approaches are dependent on the quality of the sequencing, the assembly method used, and the quality of the assembly itself (Clausen *et al.* 2016).

The grouping of ARG within each sample is not surprising, as these genes would likely increase the survival rate of the bacteria, thus representing a substantial fitness benefit and eventually becoming fixed in the population (Griffin *et al.* 2010). The fact that three out of four species of bacteria associated with BRD share many of the same ARG is likewise unsurprising, as infection is highly associated with the presence of multiple pathogens (Griffin *et al.* 2010). Additionally, previous studies have shown there is a high incidence of horizontal gene transfer of ARG-containing plasmids between *M. haemolytica* and *P. multocida* (Klima *et al.* 2014), and the presence of the integrative and conjugative elements found in these two species have been associated with isolates recovered from BRD-affected cattle (Neibergs *et al.* 2014). Here, we show that 97% of *M. haemolytica* samples and 86% *P. multocida* samples obtained from BRD-affected cattle contain regions homologous to the ICE backbone. It was interesting that no ARG were detected in five of the seven samples collected from untreated beef cattle, other than the two *P. multocida* samples. While both *M. haemolytica* and *P. multocida* are known to exist commensally in the respiratory tract of animals not affected by BRD (Neibergs *et al.* 2014), the significance of these findings needs to be substantiated by a larger number of more diverse samples.

While *in silico* analysis can be used to identify ARG within WGS data, it does not reveal the functionality or effectiveness of the ARG *in vivo*. Therefore, antimicrobial susceptibility testing should be performed alongside *in silico* methods in order to understand the expression of these ARG, at least within laboratory testing conditions. In addition, extensive metadata should be collected for each sample, including the eventual clinical outcome for the animal from which the sample was taken. These types of data would help us to understand how knowledge of the presence of ARG within a given genome could potentially predict treatment and morbidity/mortality outcomes.

### Genotype–phenotype concordance

The genotype–phenotype results from this study demonstrate the high degree of variability in concordance rates between different drugs, genes, and organisms. The ability of antimicrobial resistance genotype to predict phenotypic susceptibility has been under much discussion recently, particularly in the context of increased use of WGS as part of national food safety monitoring systems (Allard *et al.* 2016; Burrell *et al.* 2016). Some of the discordance rates presented in this study were higher than rates previously reported (Card *et al.* 2013; Agyekum *et al.* 2016). However, the available literature does not include results for the three organisms included in this study, which suggests that genotype–phenotype concordance rates may be highly species-specific. Alternatively, the breakpoints that we utilized for this study may not have been appropriate for *H. somni*, *M. haemolytica*, and *P. multocida*, especially since many of the breakpoints were not formulated for the relevant drug-bacterium-host combinations (Table 4). It has been shown that when changing the specific parameters of drug-bacterium-host, the CLSI-approved breakpoints become invalid and in cases were phenotypic resistance was seen, but no known ARG was detected, it becomes difficult to discern actual phenotypic resistance from inappropriate reporting (DeDonder and Apley 2015). However, even among breakpoints that were relevant, we found varying levels of genotype–phenotype concordance. Host- and organism-appropriate breakpoints were used for ENRO, TUL, FLOR, CHLOR, OXY, CEFT, and SPEC. While concordance rates were >98% for ENRO, OXY, and SPEC, there was significant discordance for CEFT, CHLOR, FLOR, and TUL (*P* < 0.05; Table 6). Therefore, issues concerning breakpoints do not fully account for the relatively high rates of genotype–phenotype discordance observed.

In many cases, the presence of a resistance gene correlated well with certain drugs within the relevant antimicrobial class, but not others. For instance, within *M. haemolytica* isolates the presence of the *ROB-1* gene correlated well with phenotypic resistance to AMP and PEN, but not CEFT, to which there was no phenotypic resistance. Therefore, these results suggest that *ROB-1* may not cause resistance to third-generation cephalosporins in *M. haemolytica*; a similar finding has been demonstrated with *ROB-1* in Canadian *Haemophilus influenzae* human isolates (Karlowsky *et al.* 2000). Phenotypic NEO resistance was present across all 64 isolates, and 63 of the isolates also contained *aph3-Ia*, suggesting that this gene may be a very good predictor of neomycin resistance in *H. somni*, *M. haemolytica*, and *P. multocida*.

Resistance to TYL was nearly 100% within *M. haemolytica* and *P. multocida* isolates. However, presence of the *erm42* gene was only predictive of resistance in *P. multocida* (Figure 4). Therefore, additional unrecognized mechanisms or genes are likely responsible for resistance in the other two species, although inappropriate breakpoints cannot be discounted. In contrast, the presence of *erm42* predicted TUL resistance well within *M. haemolytica*, in that all five resistant isolates contained *erm42* and none of the susceptible isolates did. However, the presence of *erm42* in all 25 *P. multocida* isolates did not result in TUL resistance (Figure 4). These results highlight the importance of considering the specific antimicrobial drug, resistance gene, organism, and host when investigating the ability of genotype to predict phenotype (or vice versa).

Results of our study demonstrate the complexity of genome-wide dynamics in mediation of resistance, and emphasize the fact that simple presence/absence of individual genes does not always predict the behavior of a given organism under varying levels of antimicrobial drug exposures. More research is needed to develop reliable and accurate genomic predictors of antimicrobial resistance, and this research will likely need to be targeted toward specific drug-bacterium-host combinations. Until such genomic predictors are identified and refined, the scientific, medical, public health, and regulatory communities should be cognizant of the potential limitations of relying solely on genomic data to guide treatment and programmatic or regulatory decisions. Such a conservative approach is especially relevant to the veterinary community, where WGS of pathogens with high animal health importance has been limited.

The sequences produced as part of this work represent a first step toward increased understanding of genome-mediated antimicrobial resistance dynamics within these important cattle pathogens. However, the variability in our results highlights the continuing criticality of collecting pertinent host, production, and health outcome information (*i.e.*, metadata) for each isolate. Without such information, the scientific community will be unable to evaluate the implications of positive identification of resistance genes within WGS data, and improve BRD morbidity/mortality outcomes by predicting the most effective treatment regime for BRD based on the specific gene-bacterium-host combination.

## Supplementary Material

Supplemental material is available online at www.g3journal.org/lookup/suppl/doi:10.1534/g3.117.1137/-/DC1.
