# Supplementary figures and images for: Whole-Genome Sequencing and Concordance Between Antimicrobial Susceptibility Genotypes and Phenotypes of Bacterial Isolates Associated with Bovine Respiratory Disease
Source: G3 (Bethesda). 2017 Jul 26;7(9):3059–71. doi: 10.1534/g3.117.1137 (PMC5592931; doi:10.1534/g3.117.1137)

## Slide 1
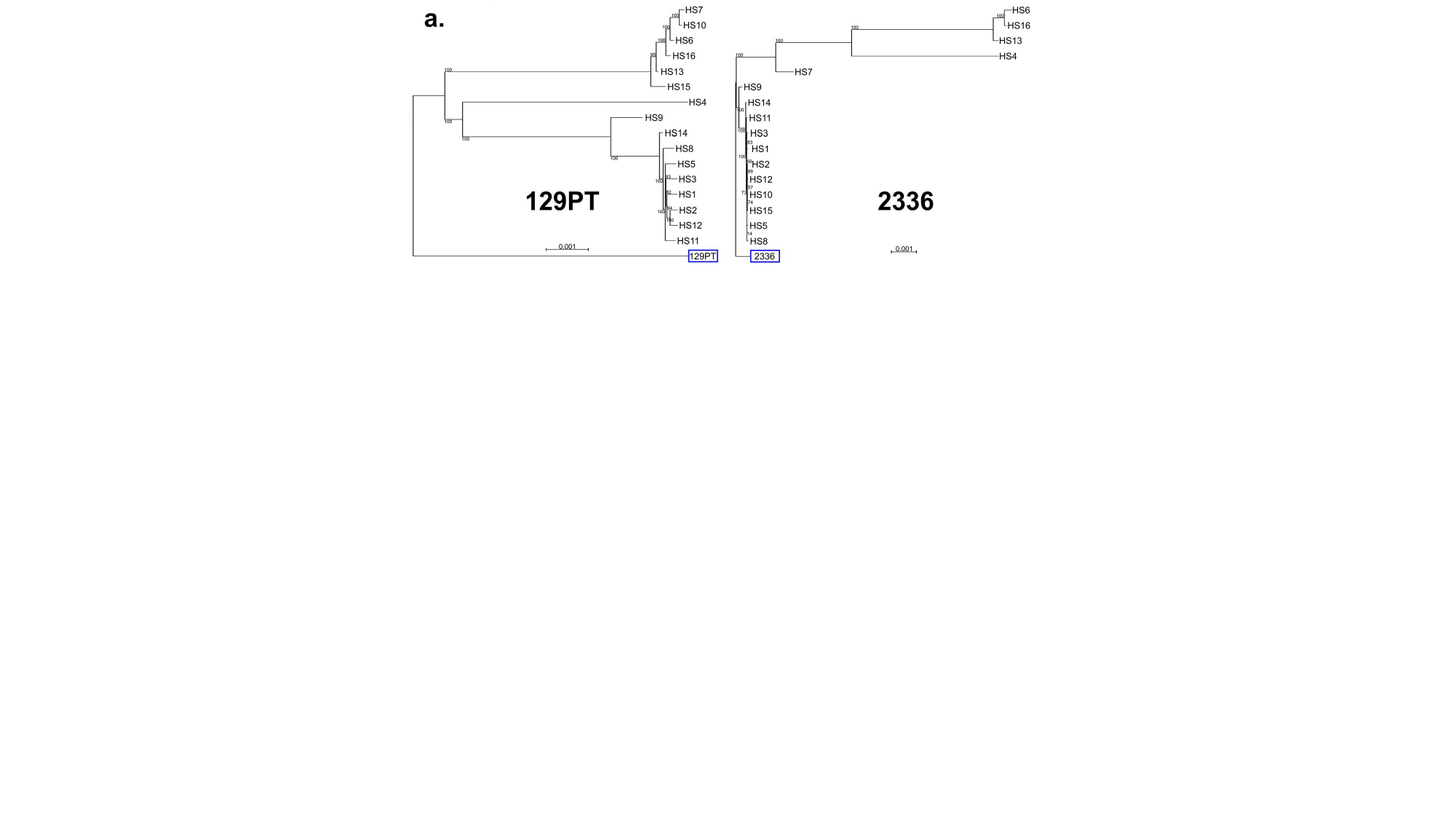

## Slide 2
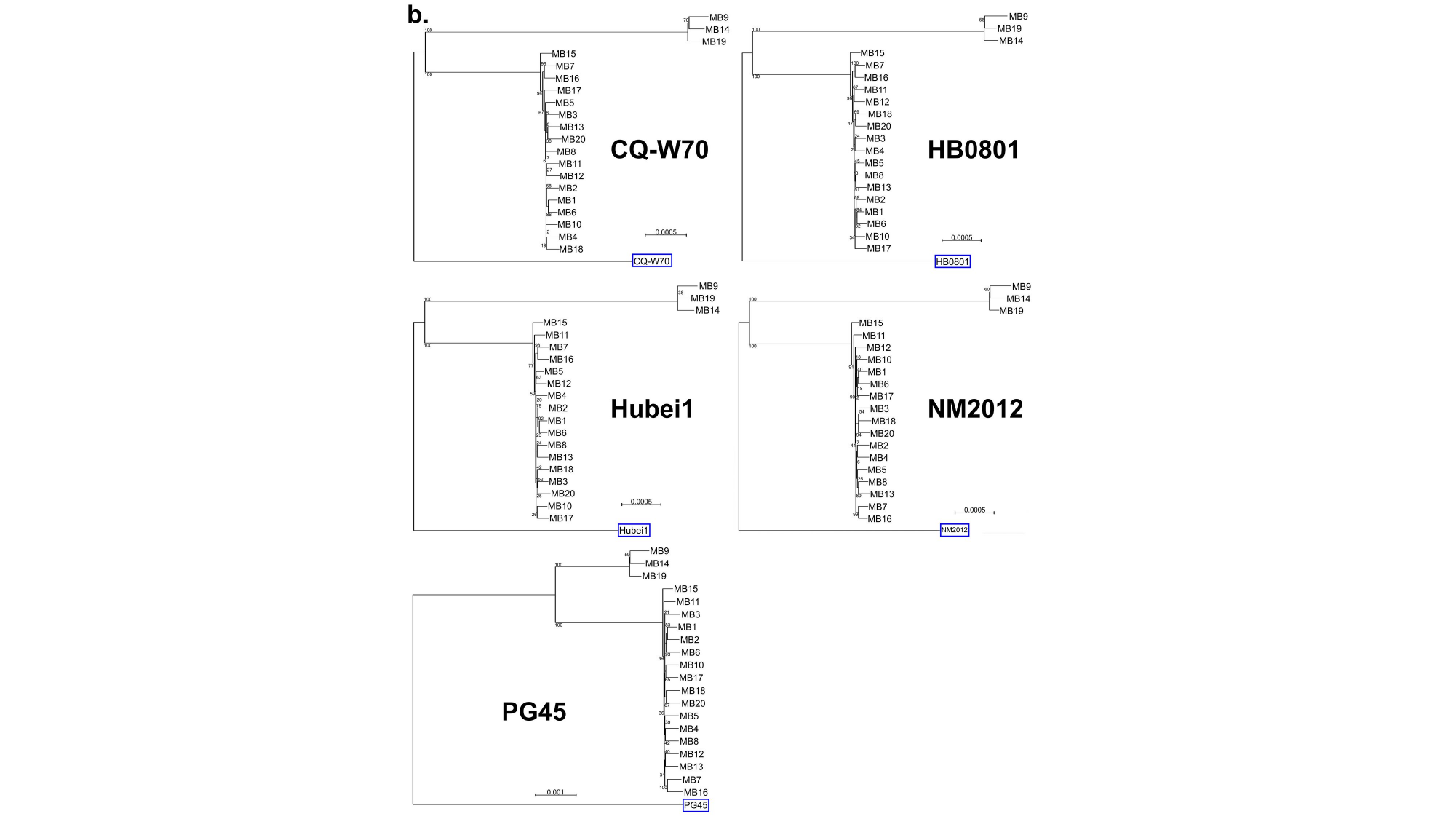

## Slide 3
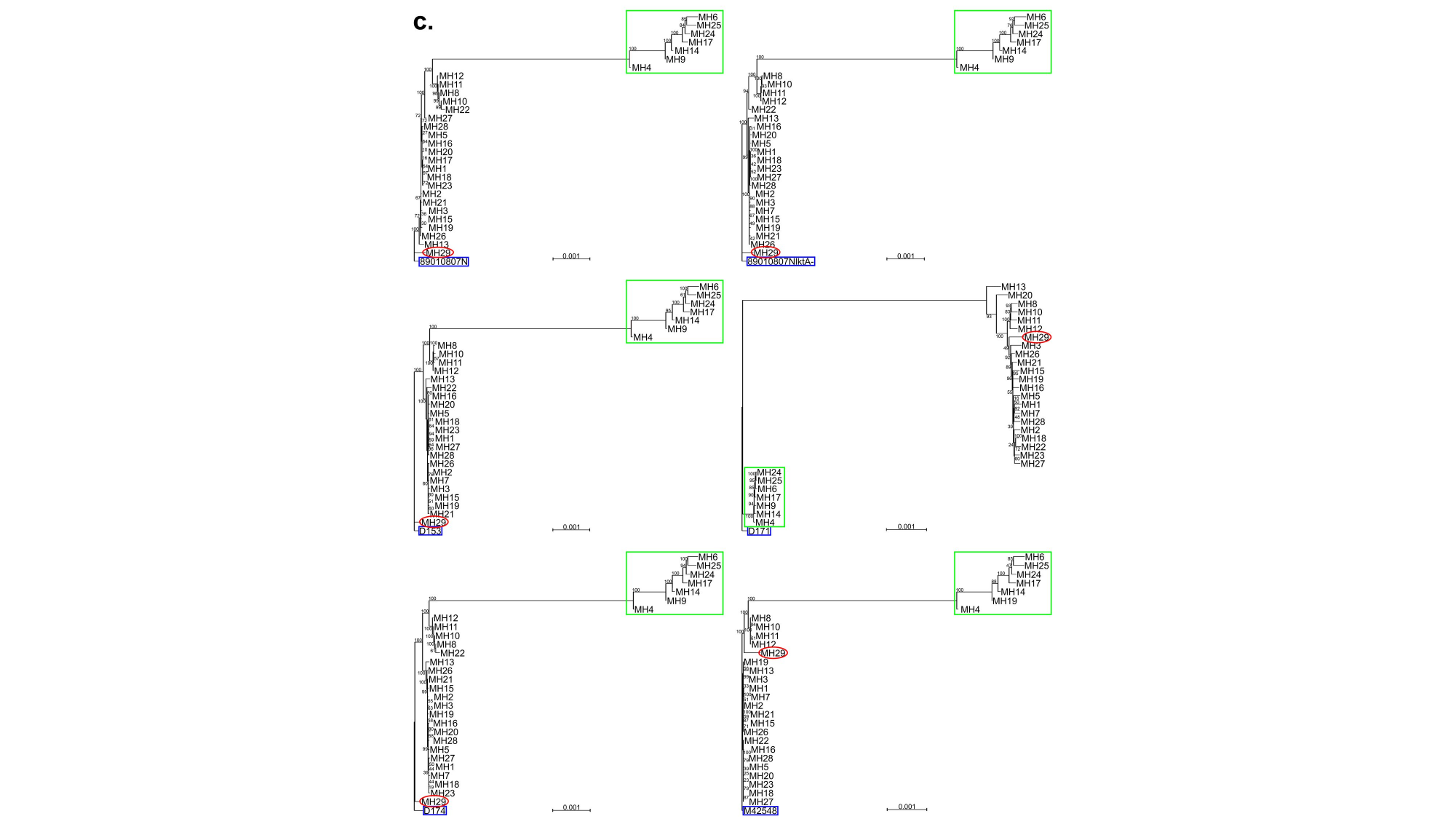

## Slide 4
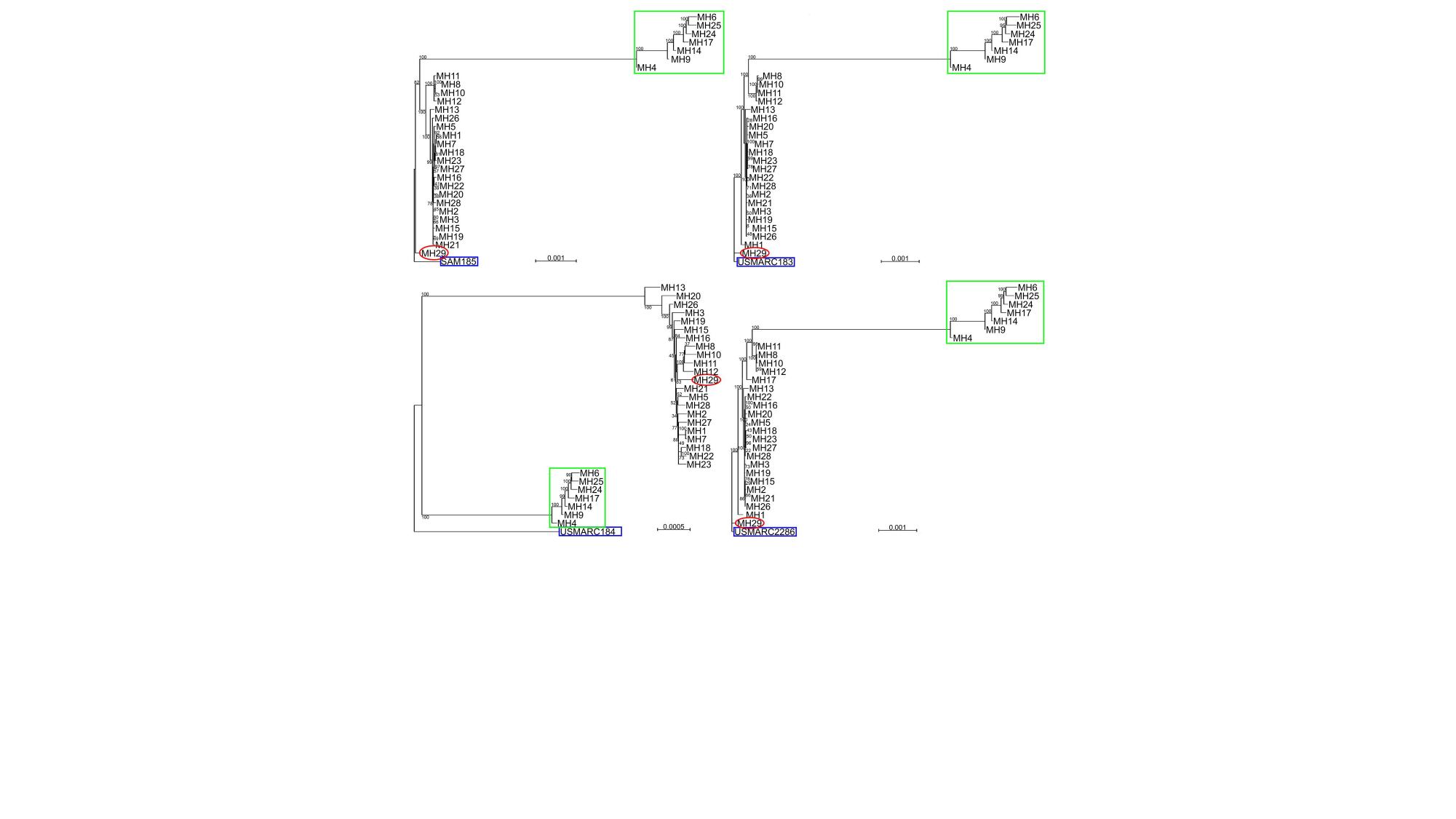

## Slide 5
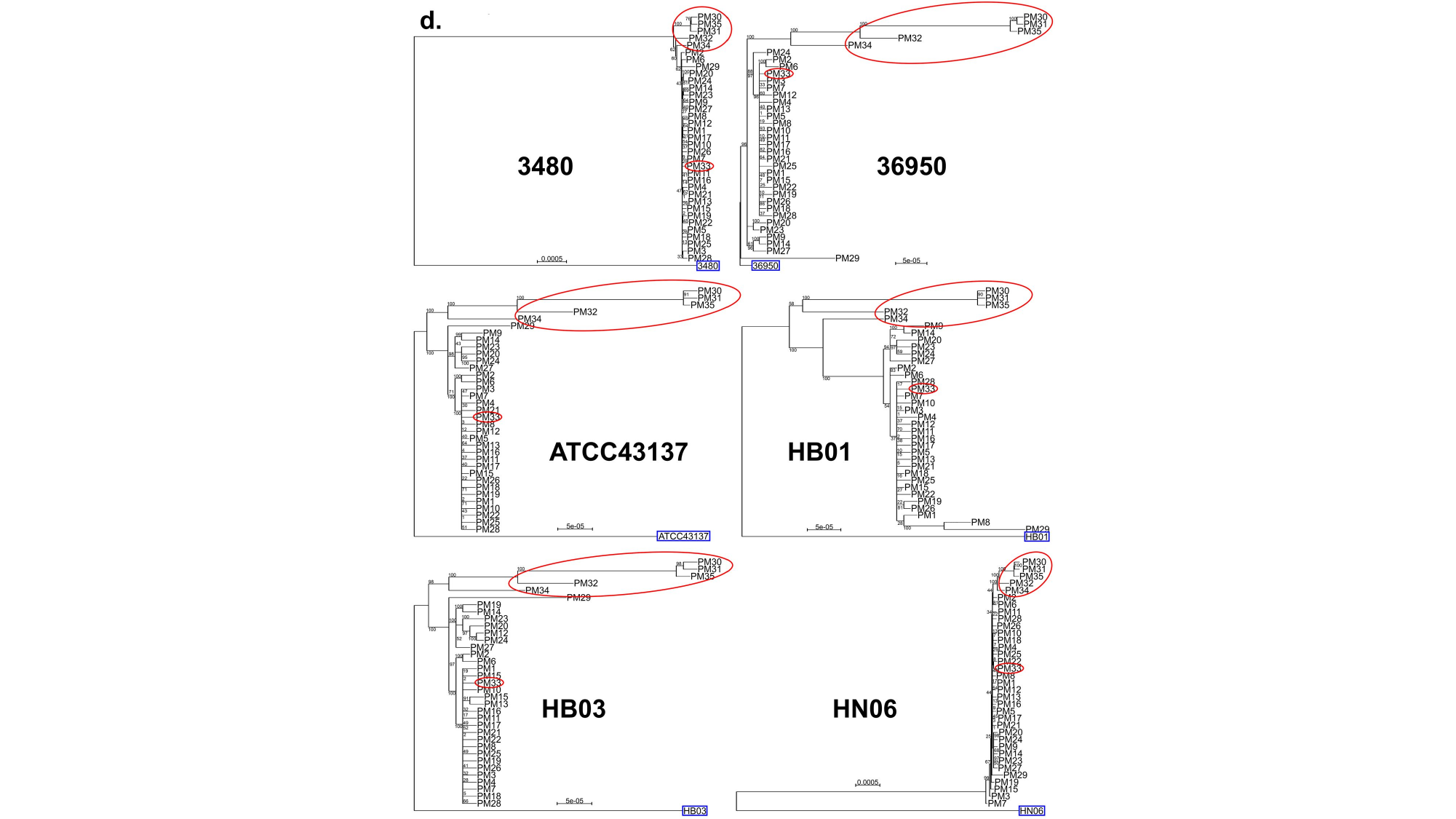

## Slide 6
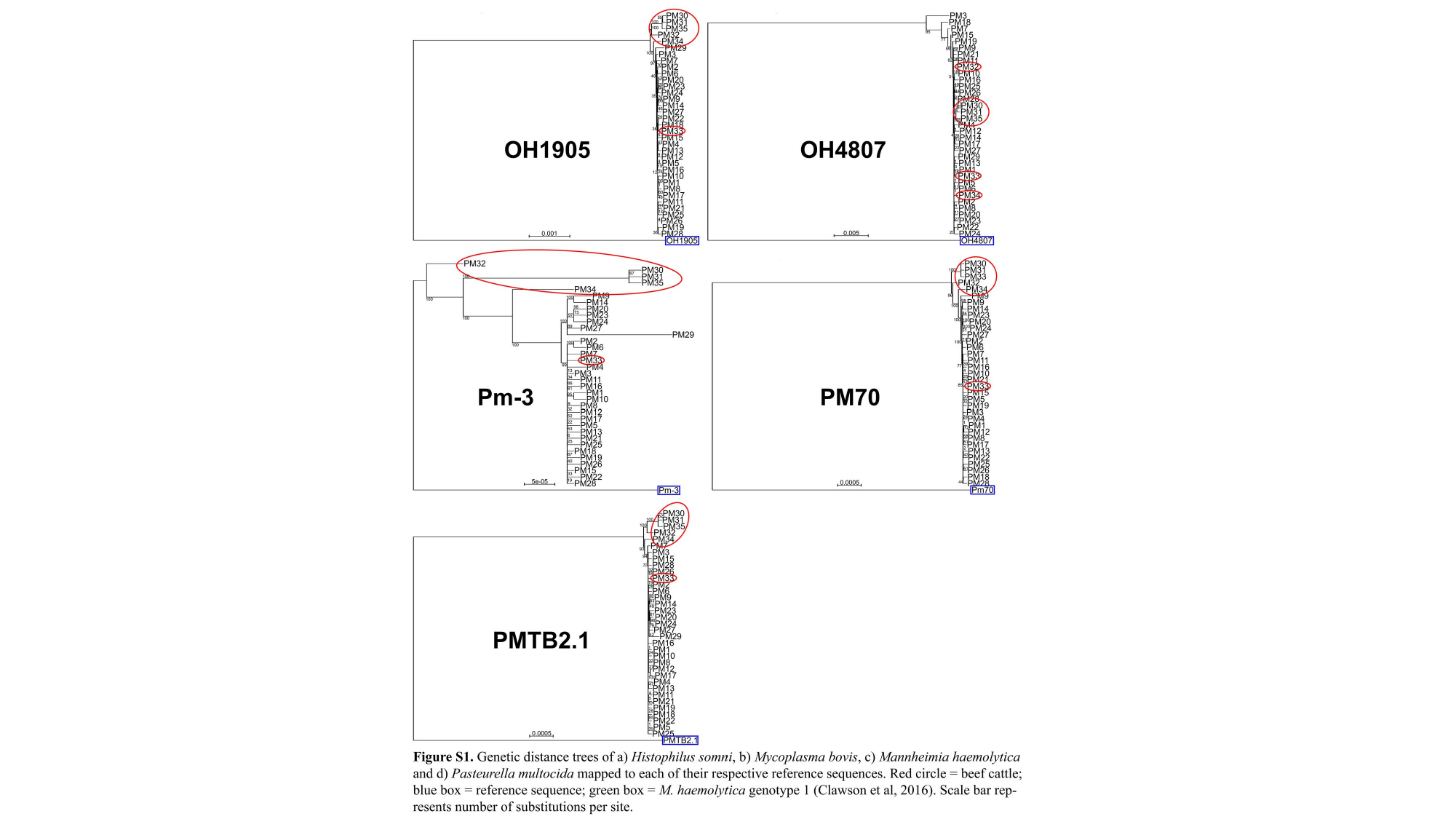

Supplement: Supplementary file 2 [file 3059FigureS1.pptx]

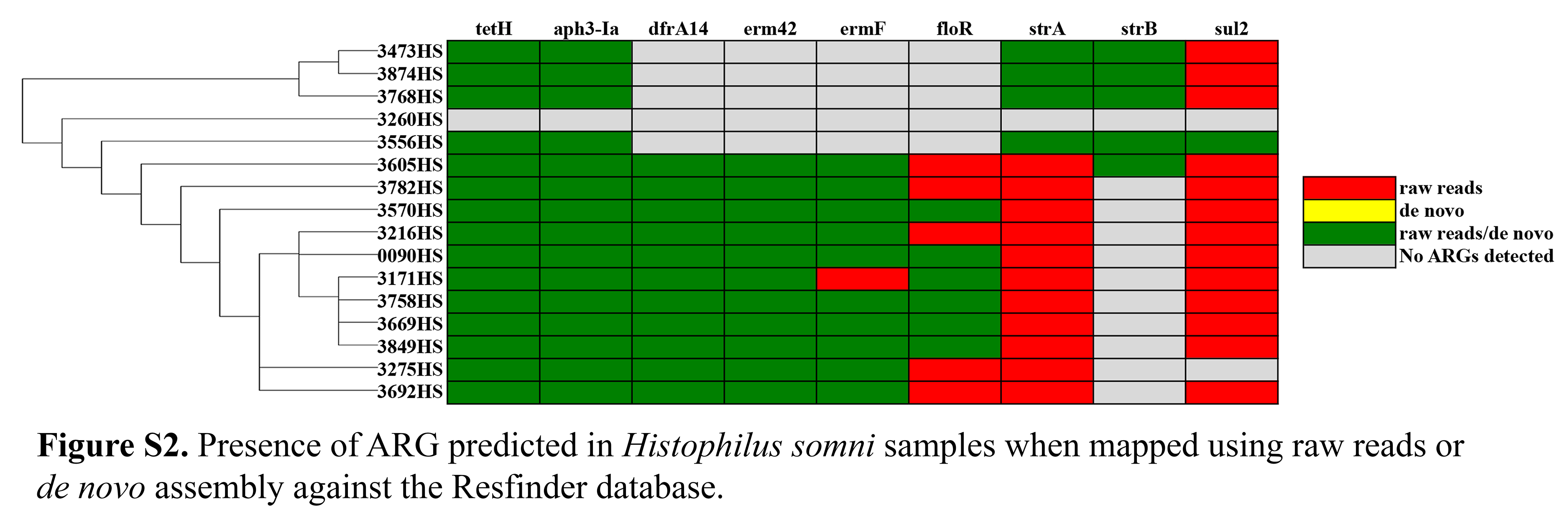

Supplement: Supplementary file 3 [file 3059FigureS2.tif]

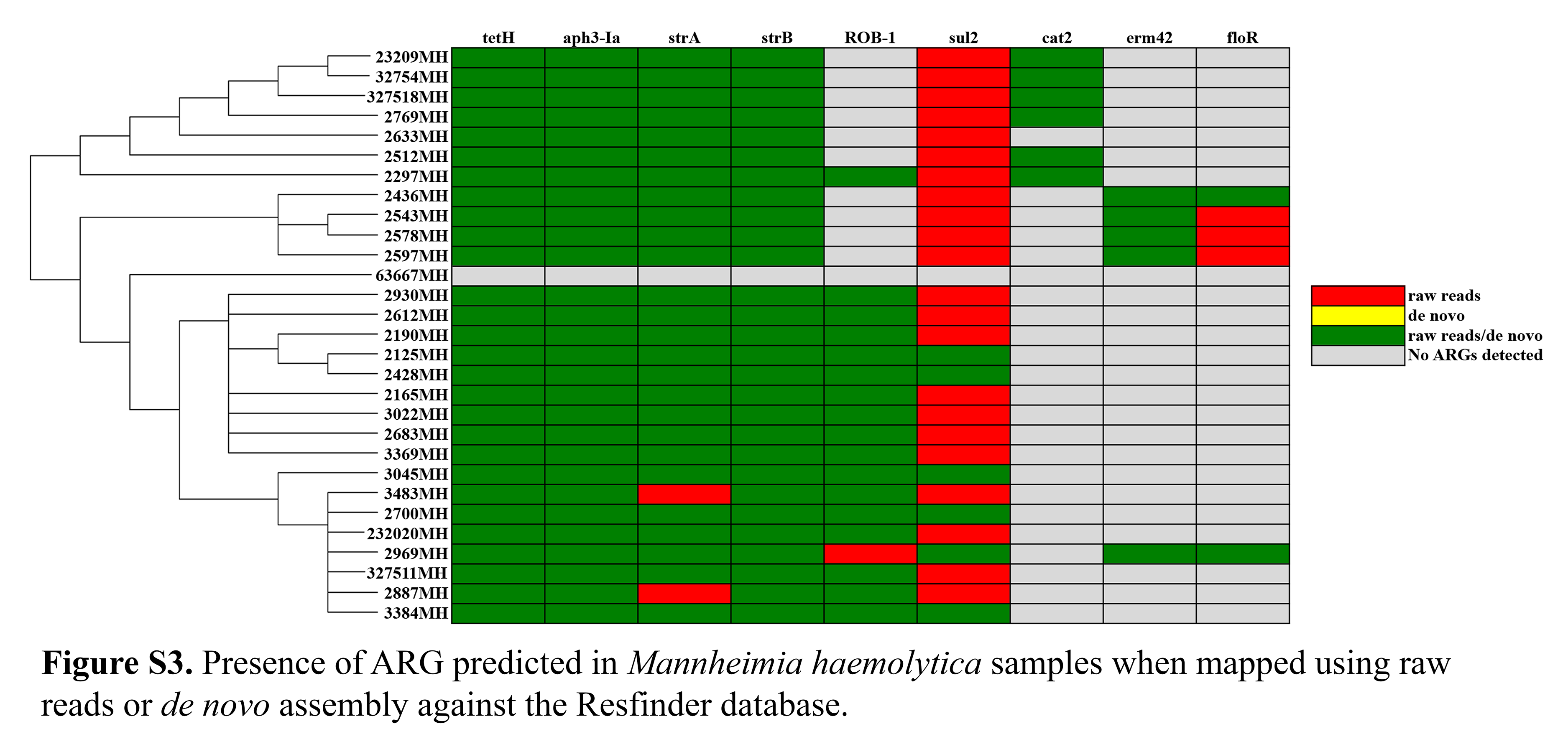

Supplement: Supplementary file 4 [file 3059FigureS3.tif]

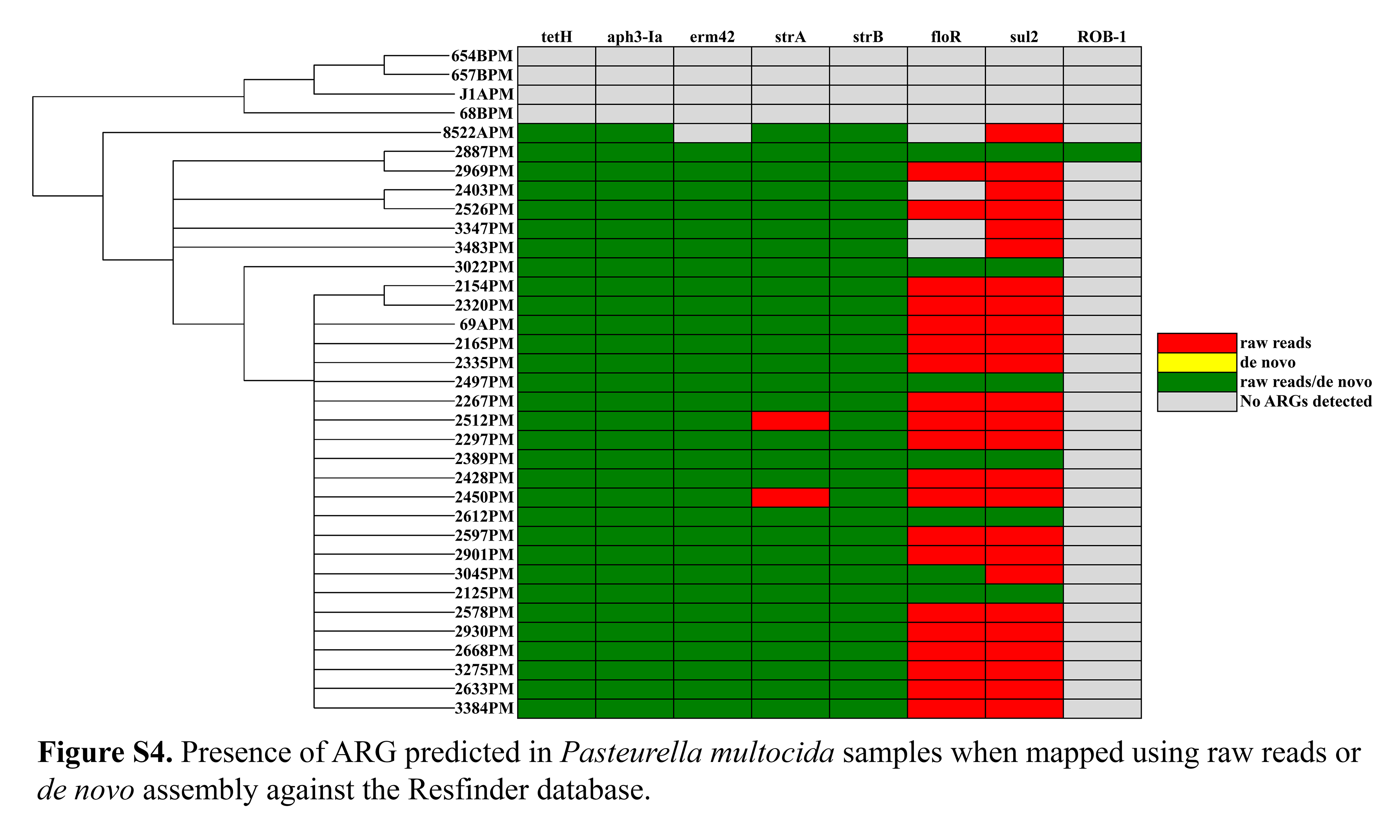

Supplement: Supplementary file 5 [file 3059FigureS4.tif]

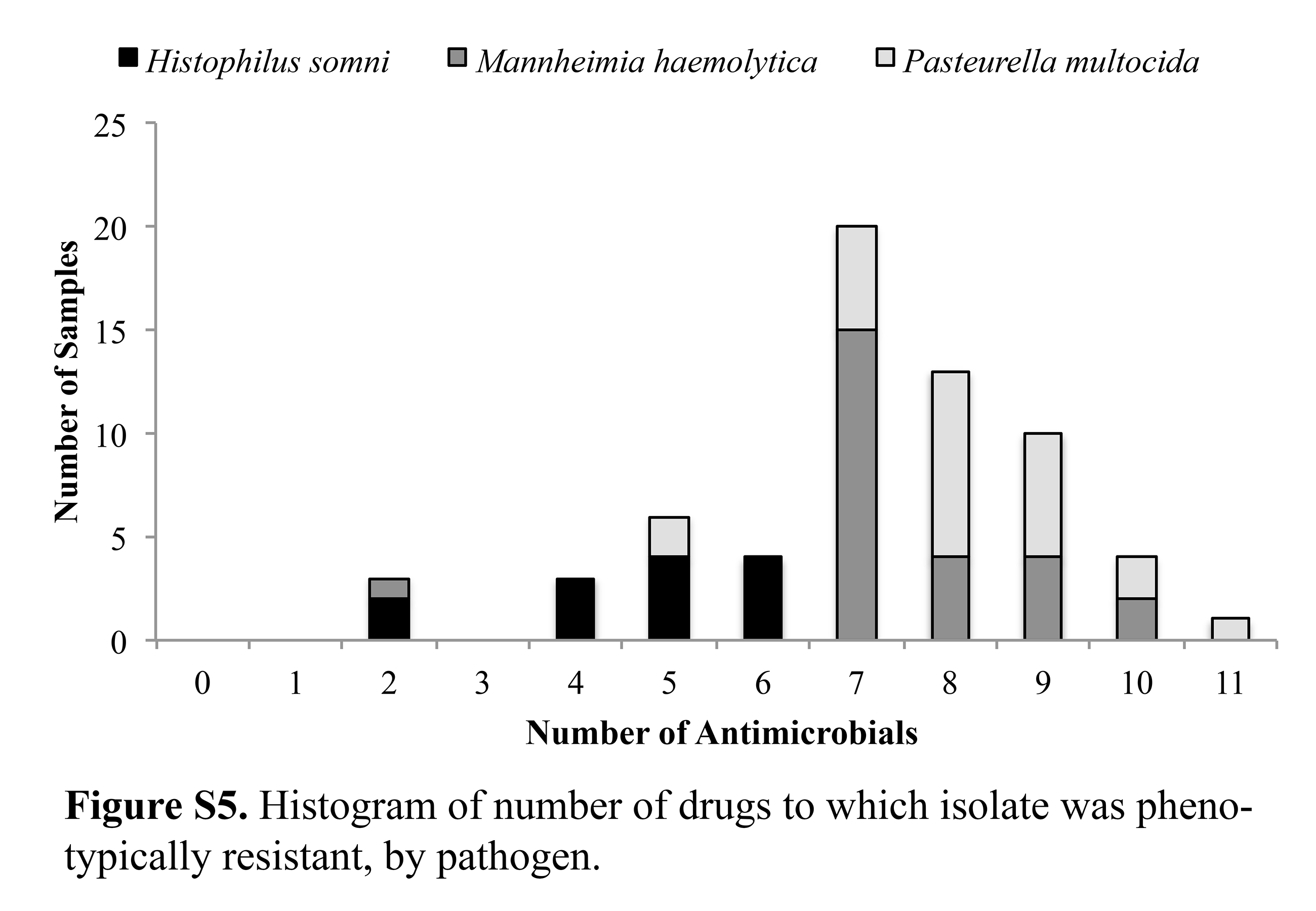

Supplement: Supplementary file 6 [file 3059FigureS5.tif]

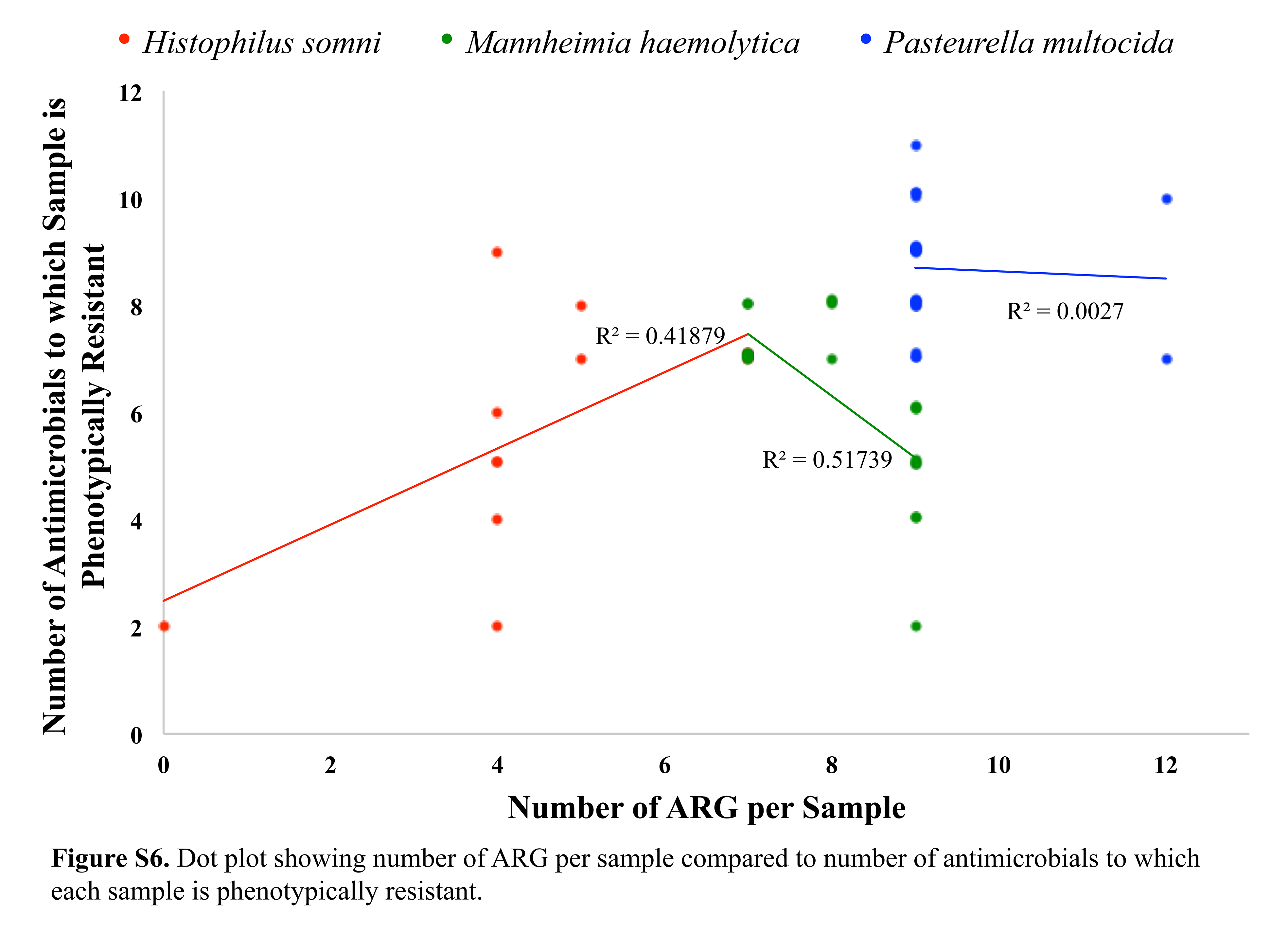

Supplement: Supplementary file 7 [file 3059FigureS6.tif]
